# Physicochemical Properties, Antioxidant Activity, and Flavor Profile of Strawberry Fruit-Based Novel Drinking Jelly Made with *Gracilaria fisheri* Seaweed as a Gelling Agent at Varying Concentrations

**DOI:** 10.3390/gels11010054

**Published:** 2025-01-10

**Authors:** Narin Charoenphun, Paramee Noonim, Somwang Lekjing, Chawakwan Nitikornwarakul, Nam Hoang Pham, Karthikeyan Venkatachalam

**Affiliations:** 1 Faculty of Science and Arts, Burapha University, Chanthaburi Campus, Chanthaburi 22170, Thailand; narinch@buu.ac.th; 2 Faculty of Innovative Agriculture, Fisheries and Food, Prince of Songkla University, Surat Thani Campus, Makham Tia, Mueang, Surat Thani 84000, Thailand; paramee.n@psu.ac.th (P.N.); somwang.s@psu.ac.th (S.L.); 3 Department of Food Science and Technology, Faculty of Agro-Industry, Kasetsart University, Bangkok 10900, Thailand; chawakwan.n@ku.th; 4 Department of Life Sciences, University of Science and Technology of Hanoi, Vietnam Academy of Science and Technology, 18-Hoang Quoc Viet, Cau Giay, Hanoi 10072, Vietnam; pham-hoang.nam@usth.edu.vn

**Keywords:** *Gracilaria fisheri*, gelling agent, drinking jelly, strawberry, physicochemical quality, flavor

## Abstract

*Gracilaria fisheri* (GF) is a red seaweed that is widely found in Southeast Asia and has gained attention for its potential bioactive compounds and versatile applications in food products. This study explored the potential of GF as a natural gelling agent in the development of sustainable strawberry-based drinking jelly. By utilizing GF at varying concentrations (0.2 (S1), 0.4 (S2), 0.6 (S3), 0.8 (S4), and 1.0% (S5)), the impact on the physicochemical, textural, phytochemical, and flavor profiles of the strawberry concentrate-based drinking jelly was examined. The results demonstrated that GF concentration significantly affected the color characteristics, structural development, and flavor profiles of the drinking jelly samples. Increasing GF levels progressively enhanced the lightness (L*) and redness (a*) values while reducing the yellowness (b*), with optimal visual appeal achieved in the S4 samples compared to others. Microscopical observations revealed that gel matrix development improved with GF concentrations up to 0.8% (S4), transitioning from a sparse, liquid-like structure at lower levels to a compact, over-gelated network at 1.0% (S5). Physicochemical parameters, including pH, total soluble solid (TSS), and TSS/titratable acidity (TA) ratios, increased with GF levels, contributing to a sweeter, less acidic product, while water activity (a_w_) decreased, enhancing jelly stability. Viscosity and sulfate content increased significantly with GF concentration, indicating improved gel strength but reduced fluidity. Phytochemical analysis revealed that ascorbic acid (AsA) and total phenolic content (TPC) decreased progressively with higher GF levels, leading to a reduction in antioxidant activity (DPPH and ABTS). Volatile compound analysis identified alcohols, esters, and aldehydes as dominant contributors to the flavor profile. 1-Octanol (waxy, citrus-like) and methyl anthranilate (grape-like, sweet) increased substantially, while minor groups such as terpenoids and phenolic compounds contributed floral and woody notes. The findings suggest that S4 samples strike the optimal balance between texture, color, flavor, and antioxidant properties, achieving a cohesive, visually appealing, and flavorful drinking jelly suitable for commercial applications.

## 1. Introduction

Macroalgae is a significant component of global aquaculture, comprising 27% of total production. Its ecological advantages and diverse applications have made it extremely popular [1]. Southeast Asia is a significant producer in this industry, underscoring the region’s critical role in cultivating and harvesting macroalgae. In addition to being an essential food source, microalgae are also important for environmental management and sustainable development, highlighting their significance for the food and aquaculture sector and larger ecological systems [2]. The genus Gracilaria is a major species of macroalgae in the phylum Rhodophyta. It is as important as red algae and has many uses in biotechnology, aquaculture, and traditional medicine. Agar production from Gracilaria species is widely used in many industries, accounting for 80% of the world’s total production [3]. Furthermore, with more than 190 species, the genus Gracilaria is widely distributed throughout tropical and temperate regions, contains high concentrations of bioactive compounds, and may have health benefits. One of the most common species in the genus is the red seaweed *Gracilaria fisheri* (GF), which has been garnered for its phenotypic plasticity and chemical diversity. These attributes support its extensive applications, rendering it highly valuable across multiple sectors, including biotechnology, aquaculture, and traditional medicine [4]. GF has long been considered a wholesome food source in Southeast Asia, Japan, and the Caribbean [5]. It is frequently used in various recipes, including salads, soups, and desserts, highlighting its cultural significance and culinary adaptability. In Thailand, it is sold either fresh or dried and mainly used to make savory salad. Apart from food, GF have long been used in traditional medicine to treat various illnesses, such as thyroid-related conditions, intestinal problems, and respiratory problems [6]. Furthermore, GF polysaccharides—particularly agar—are essential raw materials for producing a hydrocolloid widely used in the food, pharmaceutical, and biotechnology industries. The sulfated galactans, also known as agarans, from GF have remarkable gelling, stabilizing, and thickening qualities [7]. These characteristics make it possible for them to be used in various consumer products, such as desserts, soups, beverages, and jelly candies, highlighting the exceptional adaptability and worth of GF for both culinary and industrial uses. Other bioactivities that these polysaccharides display include antiviral, antibacterial, anti-inflammatory, and antioxidant qualities. The potential therapeutic effects of these bioactivities support their incorporation in nutraceuticals and functional food [8].

The fast-paced lifestyle has increased the demand for nutritious, time-efficient food options. A major challenge is developing a ready-to-eat product that satisfies consumers and satiates hunger [9]. Drinking jelly, characterized by its viscous consistency, has emerged as a novel food product with potential health benefits. Its versatility and adaptability to diverse formulations make it a convenient option for consumption at any time. Typically, the drinking jelly combines a gelatinous base with various flavorings and nutritional additives. The production and consumption of drinking jelly are influenced by its health benefits, manufacturing techniques, and sensory properties. Drinking jelly can have significant health benefits, particularly when enriched with specific ingredients. For instance, a jelly drink containing polyphenol-rich roselle calyces extract and passion fruit juice demonstrated antioxidant, anti-inflammatory, and lipid-lowering effects [10]. Producing drinking jelly involves creating a viscous base, often using ingredients such as amaranth flour or seaweed-derived gelling agents. Seaweed gelling agents are increasingly being used in the formulation of drinking jellies due to their health benefits and functional properties. Agar-agar is a natural, plant-based gelling agent. It is composed of two polysaccharides, agarose and agaropectin. In general, carrageenan and agar-agar are the common gelling agents derived from seaweed, and they are well known for their ability to form gels at low concentrations (<1%) and provide desirable texture and mouthfeel in jelly drinks [11]. Fruit juice-based drinking jelly is a versatile product that combines the nutritional benefits of fruit juice with the unique texture of drinking jelly. Strawberries are a rich source of diverse bioactive compounds, both nutritive and non-nutritive, which have been linked to various health benefits and disease prevention [12]. Strawberries, while versatile and widely used in various food applications, face significant challenges due to their short shelf life and rapid deterioration, limiting their availability as fresh fruit [13]. On the other hand, strawberry fruit concentrate is a rich source of bioactive compounds, including anthocyanins, flavonoids, and vitamin C, and provides similar health benefits as fresh strawberries and is also widely used for various food applications. There is a wide range of strawberry-based jelly products being developed [14,15]. There is limited study or information available on the utilization of strawberry concentrate and GF as a gelling agent in producing drinking jelly. Therefore, this study aims to develop a novel, sustainable, and nutrient-rich drinking jelly product using strawberry concentrate and GF as a natural gelling agent, and it is achieved by varying the concentration of GF in the formulation; furthermore, this study has investigated the impact on the physicochemical, antioxidant, and flavor properties of the drinking jelly.

## 2. Results and Discussion

### 2.1. Color Characteristics and Appearance

**Figure 1:**
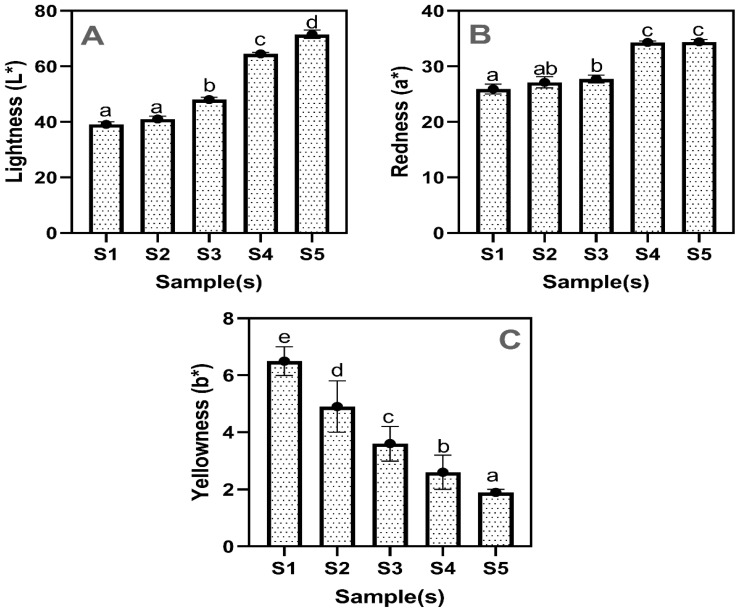
Color characteristics (Lightness (L*) (**A**), Redness (a*) (**B**), and Yellowness (b*) (**C**)) of drinking jelly made of strawberry fruit concentration and different concentrations of GF gelling agent. Note: Different letters of the alphabet in the figure show significant differences. S1–S5 represent the drinking jelly samples prepared with varying concentrations of GF gelling agent: S1 (0.2%), S2 (0.4%), S3 (0.6%), S4 (0.8%), and S5 (1.0%).

Color is a critical sensory attribute in food product development, significantly influencing consumer perception, acceptability, and purchasing behavior [16]. Hydrocolloids, such as seaweed-derived gelling agents, play a pivotal role in modifying food matrix properties, including chromatic characteristics [17,18]. The results of color characteristics, including lightness (L*), redness (a*), and yellowness (b*) of drinking jelly made with strawberry fruit concentrate and varying concentrations of GF gelling agent, are shown in Figure 1A–C. The L* values of the drinking jelly samples exhibited a continuous increase, and, overall, the increased concentration of gelling agent had effectively increased the L* value in the samples; however, at lower concentrations, no significant differences were observed. The S1 and S2 samples showed L* values around 40 with no significant differences, whereas the S3–S5 samples showed L* values of 50, 65, and 70, respectively. This indicates that adding GF gelling agent to the drinking jelly progressively increases the lightening effect. Manurung et al. [19] reported that adding a seaweed-based gelling agent improved the lightness of the jelly by altering the moisture and sugar content. Faridah [20] explored that adding a seaweed gelling agent in the jelly enhanced the color characteristics and overall appeal. Similarly, the a* values in the drinking jelly samples were constantly increased with the GF gelling agent’s increased concentration. The S1 samples displayed an a* value of 26, while the S2 samples showed slightly higher a* values (27) and, as the concentration increased, the a* values in the S3–S5 samples increased to 27.7, 34.3, and 34.4, respectively. This indicates a significant increase in the a* value in the samples at higher concentrations of GF gelling agents. Jayasinghe et al. [21] reported that natural pigment in the seaweed could contribute to the jelly’s color. Trilaksani et al. [22] suggested that the increase in color characteristics is the synergistic effect of both the pigment’s natural color and the other jelly ingredients. This is in accordance with the present study, where strawberry fruit concentrate and GF gelling agent contribute to red pigments. On the other hand, the b* values in drinking jelly continuously decreased as the GF gelling agent concentration increased. 

**Figure 2:**
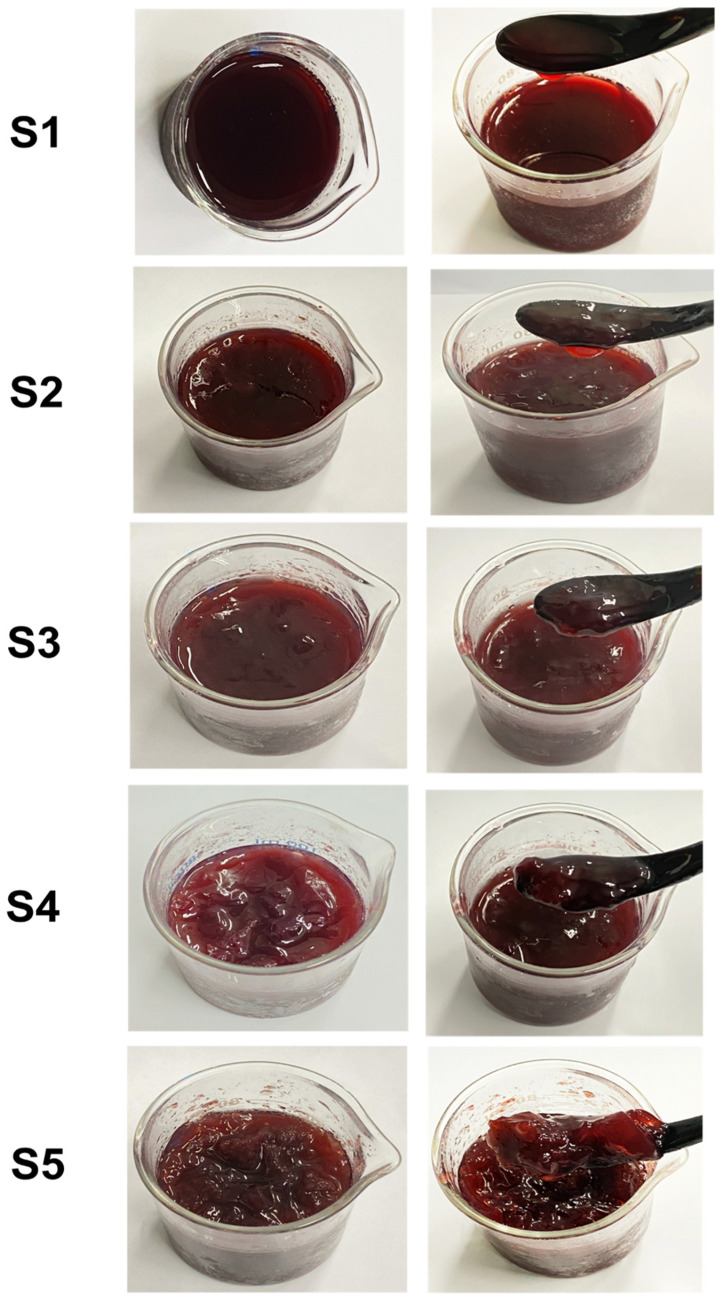
Visual appearance of the drinking jelly made with strawberry fruit concentrate and different concentrations of GF gelling agent. Note: S1–S5 represent the drinking jelly samples prepared with varying concentrations of GF gelling agent: S1 (0.2%), S2 (0.4%), S3 (0.6%), S4 (0.8%), and S5 (1.0%).

Among the sample variants, the S1 sample had the highest b* value (6.5), followed by the others, and the S5 sample had the lowest b* value (1.9). The other samples ranged from 2.6 to 4.9 (*p* < 0.05). This indicates that higher concentrations of the GF gelling agent result in a less yellow hue in the drinking jelly. Hubbermann [23] reported that using different natural pigments affects the color properties of jelly, certain that elevated levels of red pigment in the jelly could adversely reduce the yellowness. Overall, the color characteristics results indicate that the concentration of the GF gelling agent significantly affects the color attributes of drinking jelly. Increased concentrations of the gelling agent lead to a lighter, redder, and less yellow jelly. This is in accordance with the study of Dalabasmaz et al. [24] and Kim et al. [25]. Additionally, the change in color characteristics can be attributed to the interaction between the seaweed gelling agent and the strawberry fruit concentrate, which may influence the overall visual appeal of the product. Among the different concentrations, the optimal concentration of GF gelling agent appears to be around 0.8% (S4), where the jelly achieves a desirable balance of lightness and redness, enhancing its aesthetic appeal. Figure 2 shows the appearance of a strawberry-based drinking jelly made using different concentrations of GF gelling agent.

The pictorial reference showed that increasing the GF concentration in the drinking jelly composition significantly affected the consistency and homogeneity of the drinking jelly. The S1–S2 samples appeared liquid-like, with some visible stratification, indicating a low gelation level. On the other hand, by increasing the gelling agent concentration to 0.6–0.8% (S3–S4), the jelly became more cohesive, showing no phase separation with a homogenous distribution. On the contrary, increasing the concentration to 1.0% resulted in very rigid jelly that was too dense, breaking the desired fluidity in drinking jelly. Sagril et al. [26] reported that an increase in gelling agent concentration results in a denser network, enhancing the gel’s mechanical properties and stability by increasing the interaction among the gelling agent molecules and strengthening the network structure.

### 2.2. Microscopical Observations

**Figure 3:**
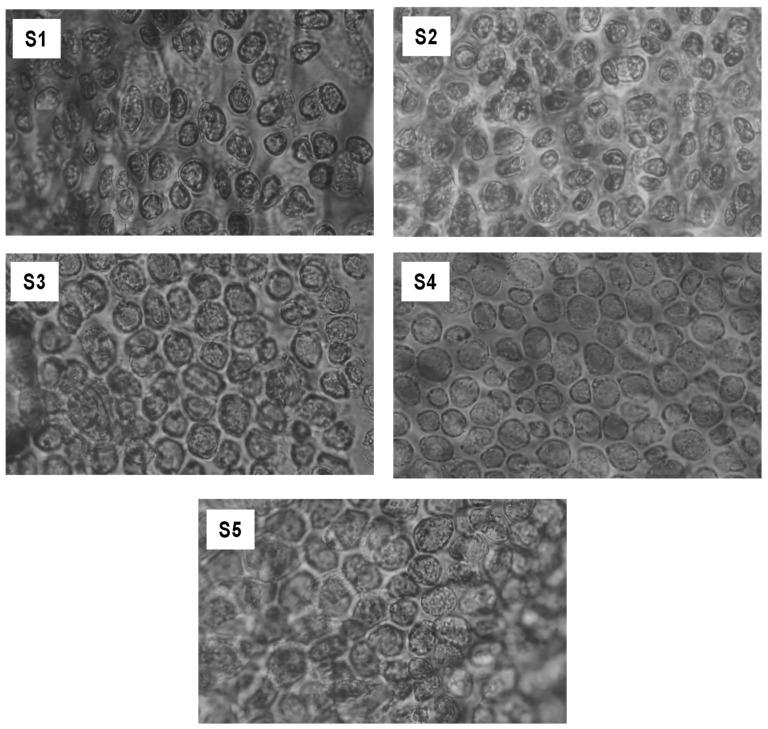
Microscopical observation of drinking jelly made with strawberry fruit concentrate and different concentrations of GF gelling agent. Note: S1–S5 represent the drinking jelly samples prepared with varying concentrations of GF gelling agent: S1 (0.2%), S2 (0.4%), S3 (0.6%), S4 (0.8%), and S5 (1.0%).

Microscopic observation of drinking jelly can provide insights into its physical properties, such as texture, viscosity, and structural composition [27]. The microscopic observations of drinking jelly made of strawberry fruit concentrate with varying concentrations of GF gelling agent are shown in Figure 3. The results indicate that gelling agent concentration is key in developing high-quality drinking jellies. The structural development of the gel matrix in the drinking jelly samples increased with higher concentrations of GF gelling agent. The S1–S2 samples formed a sparse and poorly developed gel network characterized by insufficient molecular cross-linking and loose interconnectivity, resulting in a liquid-like consistency. Hanabusa and Suzuki [28] reported that a gelling agent at lower concentrations in jellies may not form a sufficiently dense network, leading to poor visibility and a loose gel matrix under microscopic observations. Meanwhile, the S3–S4 samples containing higher levels of GF gelling agent showed a well-defined and interconnected gel matrix, optimal homogeneity, and tightly packed structures. Increasing the concentration of GF in the drinking jelly continuously produced a denser matrix with reduced interstitial spaces, presenting a more compact gel structure. However, at 1% (S5), the drinking jelly became over-gelated, with minimal interstitial spaces and a rigid matrix, which substantiated visual observations of reduced drinkability. Studies have reported that higher concentrations of polysaccharide-based gelling agents enhance the structural properties of gel matrices, creating a denser network that improves elasticity. Increased viscosity reduces flowability, and better water retention enhances stability and firmness; however, it reduces the applicability of the gelling agent in drinking jelly [29,30,31]. Overall, the results evidence that GF gelling agent at concentrations below 0.6% could not achieve appropriate gelation and thus resulted in a weak matrix with poor texture. At 0.8%, the gel network was homogeneous and continuous, producing a desirable texture and attractive appearance. While concentrations above 0.8% gradually reduced the fluidity of the jelly, a 1% GF gelling agent resulted in an increasingly firm texture unsuitable for drinking jelly.

### 2.3. pH, TSS, TA, and TSS/TA Ratio

**Figure 4:**
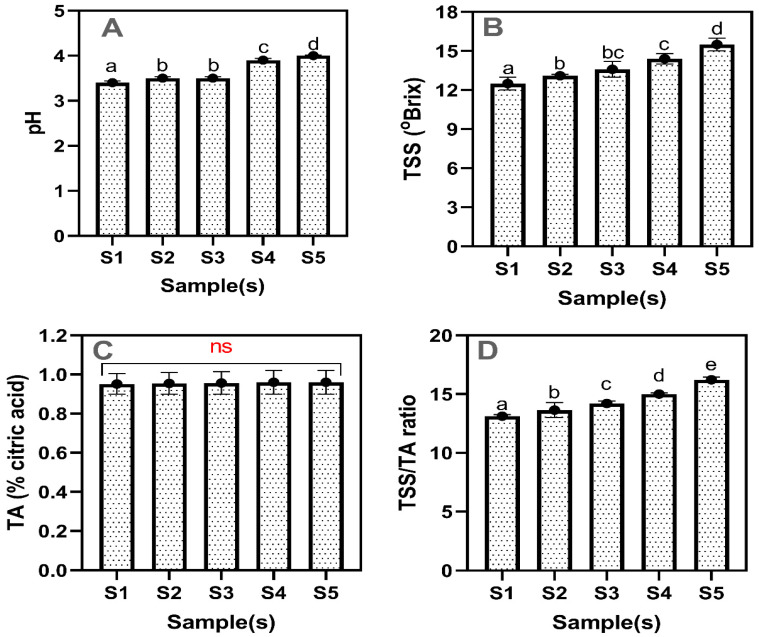
Change in pH (**A**), TSS (**B**), TA (**C**), and TSS/TA ratio (**D**) ratio of drinking jelly made of strawberry fruit concentrate and different concentrations of GF gelling agent. Note: Different letters of the alphabet in the figure show significant differences, and ns represent the non-significant. S1–S5 represent the drinking jelly samples prepared with varying concentrations of GF gelling agent: S1 (0.2%), S2 (0.4%), S3 (0.6%), S4 (0.8%), and S5 (1.0%).

Figure 4 shows the changes in the pH, TSS, TA, and TSS/TA ratio of the drinking jelly made with different concentrations of GF seaweed gelling agent. Overall, the pH values of the tested drinking jelly samples ranged from 3.4 to 4.0 (Figure 4A). Taub et al. [32] reported that a pH below 5 is generally effective for preserving jellies as it inhibits the growth of common spoilage organisms. If the pH is increased, the chance of microbial growth will increase, resulting in a shorter shelf life of the drinking jelly. The S1 samples had the lowest pH value, while the S5 samples had the highest (*p* < 0.05). The results showed that the pH values of the S1 and S2 samples were significantly lower than those of the S4 and S5 samples. Furthermore, the S3 samples exhibited intermediate pH values. This variation in pH levels indicates a range of acidity among the samples, with S1 being the most acidic and S5 being the least acidic. Gani et al. [33] reported that an increased concentration of gelling agent from seaweed in drinking jelly increases the pH levels, leading to a less acidic environment in the jelly matrix. Lee et al. [34] reported that gelling agents derived from seaweed are alkaline because they contain sulfate groups, which can ionize in solution. This ionization increases the pH and alters the charge interactions between the gelling agent molecules and water. The TSS values in the drinking jelly ranged from 12.5 to 15.1 (Figure 4B). The addition of the GF gelling agent in the drinking jelly significantly affected the TSS level (*p* < 0.05). The S1 sample had the lowest TSS value, whereas the S5 sample had the highest. The S2–S4 samples showed a gradual increase in TSS levels as the GF concentration in the drinking jelly composition increased. However, the difference in the TSS increment between the S2 and S3 samples was minimal. Gani et al. [33] and Perwira [35] reported that an increased concentration of seaweed-based gelling agent in the drinking jelly could influence the TSS levels by altering the water retention and solution distribution within the gel. Acidity plays a key role in determining the taste and stability of the jelly, as more acidic products tend to have a tangier taste and potentially longer shelf life [36]. The TA value of the drinking jelly samples is shown in Figure 4C. The TA values were consistent across all samples (*p* ≥ 0.05), ensuring the jelly maintained a uniform acid level. Presumably, the TA values in the drinking jelly were primarily contributed by the strawberry concentrate, as strawberries contain a moderate level of organic acids, ranging from 0.8% to 2.0% [37]. The stable levels of TA are due to the unchanged proportion of strawberry concentrate in the drinking jelly compositions. Generally, the consistent TA values suggest that the differences in pH and TSS are not due to variations in citric acid content. The TSS/TA ratio is an important indicator and a critical parameter in jelly production, influencing the final product’s taste and texture. The TSS/TA ratio ranged from approximately 13 to 16 (Figure 4D). Increasing the GF concentration in the drinking jelly composition effectively increased the TSS/TA ratio. The S1 samples exhibited the lowest TSS/TA ratio, while the S5 samples had the highest ratio. A higher TSS/TA ratio generally indicates a sweeter, more palatable product [38].

### 2.4. Moisture, a_w_, Viscosity, Sulfur Content, Total, and Reducing Sugar Contents

**Figure 5:**
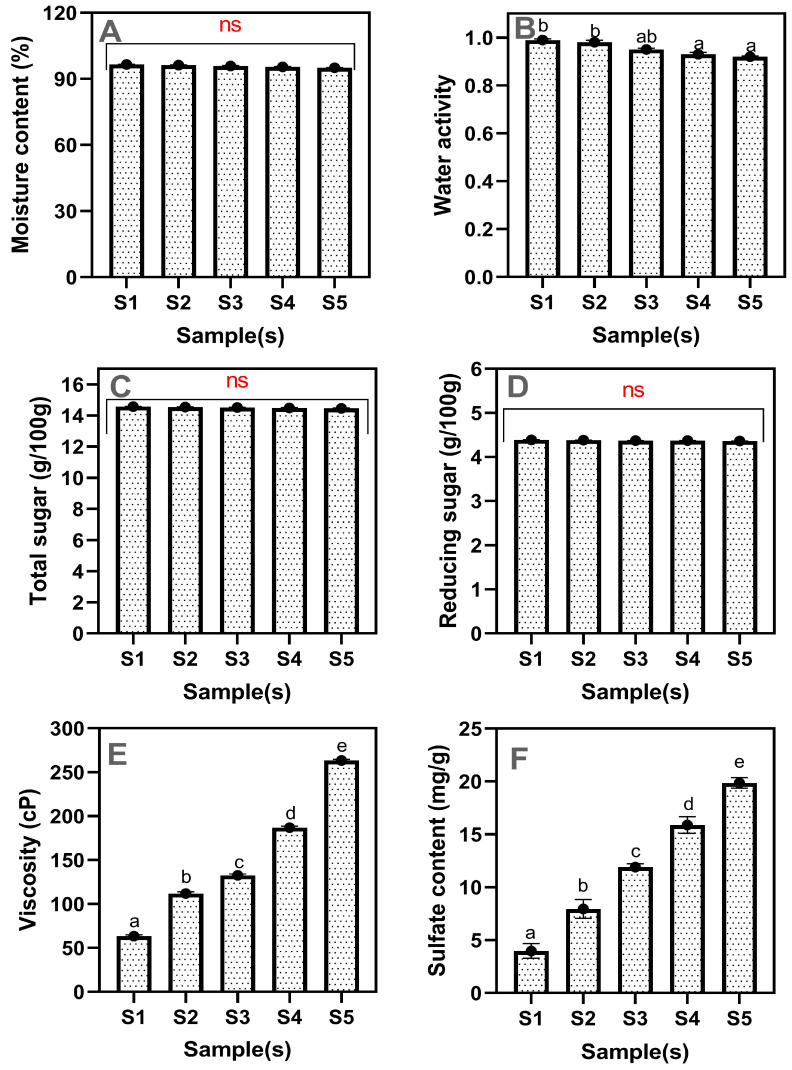
Changes in moisture content (**A**), water activity (**B**), total sugar (**C**), reducing sugar (**D**), viscosity (**E**), and sulfate content (**F**) in the drinking jelly made of strawberry fruit concentrate and different concentrations of GF gelling agent. Note: Different letters of the alphabet in the figure show significant differences, and ns represent the non-significant. S1–S5 represent the drinking jelly samples prepared with varying concentrations of GF gelling agent: S1 (0.2%), S2 (0.4%), S3 (0.6%), S4 (0.8%), and S5 (1.0%).

The changes in the moisture content, a_w_, total sugar, reducing sugar, viscosity, and sulfate content of the strawberry fruit concentrate-based drinking jelly made of varying concentrations of GF gelling agent are shown in Figure 5A–F. Moisture content is critical, as it ensures uniform hydration across all samples, essential for maintaining product quality and shelf life [39]. Non-significant differences were observed in the moisture content of the drinking jelly samples despite the variants (Figure 5A). The moisture content in the drinking jelly ranged between 95.04 and 96.52. The overall trend of moisture content in the drinking jelly was in decrement. S1 samples had slightly higher moisture than the S4–S5. Generally, the gelling agent forms a network that entraps the water molecule within its structure. The ability of this network to retain water is more dependent on its structural integrity rather than the concentration of the gelling agent [40,41]. This could be the reason why increased GF concentration did not impact the moisture content in the drinking jelly. The controlled level of a_w_ contributes to the preservation of the jelly by inhibiting growth and extending the shelf life of semi-wet food products [42,43]. The present study showed that the a_w_ level in drinking jelly decreased steadily with increased GF gelling agent concentration. Compared with moisture content, the a_w_ in drinking jelly significantly decreased and was influenced by the GF gelling agent concentrations. The S1 sample showed a high a_w_ level of 0.99, whereas the S2, S3, and S4 samples showed intermediate a_w_ levels of 0.98, 0.95, and 0.93, respectively, and the S5 sample showed the lowest a_w_ level of 0.92. The present study a_w_ range (0.92–0.99) was at the standard level for drinking jelly or fruit jelly-based products. This is in accordance with the study of Rittisak et al. [9]. Generally, seaweed polysaccharide-based gelling agents such as agar, carrageenan, and alginate have a high water-binding capacity, which contributes to the reduction of a_w_ in jellies, and this is due to their ability to form a gel matrix that entraps water molecules [44]. On the other hand, the total sugar and reducing sugar levels did not change in the drinking jelly samples despite the GF concentration variations, indicating that the gelling agent did not influence the sugar content in the drinking jelly (Figure 5C, D). However, when comparing the reduced sugar, the total sugar content was high, almost double the amount of reduced sugar. The higher total sugar content in drinking jelly than in reducing sugar is due to the dominance of non-reducing sugars such as sucrose in the formulation. The consistent levels of total sugar and reducing sugar in the samples contribute to a consistent taste profile and uniform flavor. Viscosity plays a critical role in drinking jelly’s formulation and properties by impacting texture, stability, sensory appeal, and overall functionality [45]. The viscosity levels in the drinking jelly continuously increased with the GF gelling agent concentrations (Figure 5E). The viscosity levels in the drinking jelly ranged between 63.40 and 263.3 cP. This is in accordance with the study of Pratiwi et al. [46]. The S5 samples showed a higher viscosity, gradually decreasing as the GF concentration decreased; the S1 samples showed the lowest viscosity levels. The increase in viscosity indicates a thicker and more gel-like consistency, which can enhance the texture and mouthfeel of the product [47]. Similarly, the sulfate content varied considerably among the samples, ranging from 3.97 to 19.85; S1 had the least sulfate content, whereas the S5 sample had the highest sulfate content (Figure 5F). Seaweed gelling agents often contain sulfate groups due to their natural chemical structure, which plays a crucial role in the gelling and functional properties [48]. The increase in sulfate content with higher concentrations of the GF gelling agent likely enhances the ionic interactions within the gel network, contributing to a stronger and more stable gel structure [49]. Overall, the results indicate that varying the concentration of GF seaweed gelling agent significantly impacts the a_w_, viscosity, and sulfate content of the drinking jelly. In contrast, the moisture content, total sugar, and reducing sugar levels remain consistent.

### 2.5. Textural Properties

**Figure 6:**
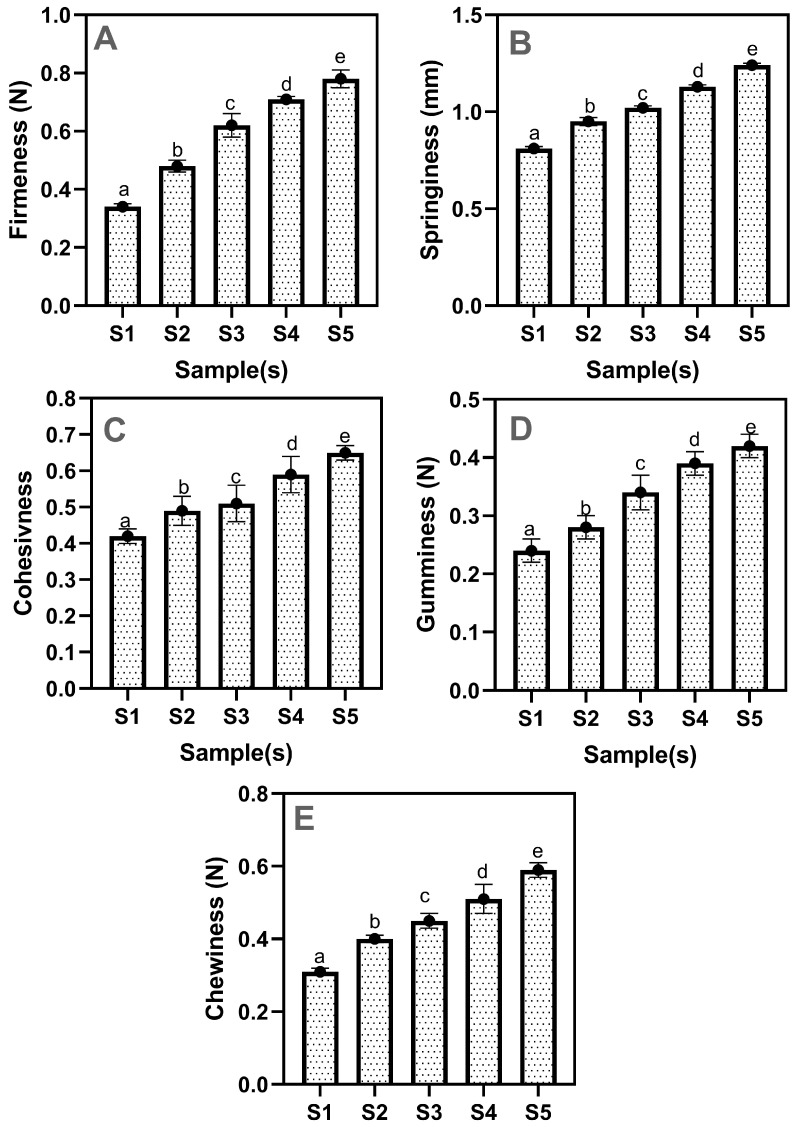
Textural properties (Firmness (**A**), Springiness (**B**), Cohisiveness (**C**), Gumminess (**D**), and Chewiness (**E**)) of drinking jelly made of strawberry fruit concentrate and different concentrations of GF gelling agent. Note: Different letters of the alphabet in the figure show significant differences. S1–S5 represent the drinking jelly samples prepared with varying concentrations of GF gelling agent: S1 (0.2%), S2 (0.4%), S3 (0.6%), S4 (0.8%), and S5 (1.0%).

The textural property of drinking jelly is normally influenced by the gelling agent type and concentration, pH, ionic strength, and storage conditions [50]. The textural properties of drinking jelly, such as firmness, springiness, cohesiveness, gumminess, and chewiness, were tested; the results are shown in Figure 6A–E. Overall, the results showed that the addition of varying concentrations of GF gelling agent in the drinking jelly composition significantly affected the textural properties (*p* < 0.05). Garrido et al. [51] reported that variation in gelling agent concentration significantly influenced the textural properties, including gel strength, cohesiveness, and firmness in the fruit-based jelly. The firmness values of the drinking jelly ranged from 0.34 N to 0.78 N, and the increased concentration of GF gelling agent, particularly in the S4 and S5 samples, produced a firmer texture in the drinking jelly compared to lower concentrations (S1–S3); however, the lower concentrations did not provide the right consistency for the drinking jelly firmness. A similar finding was also noted by Afriani [52]. This study reported that the firmness value in the drinking jelly increased proportionally with the gelling agent concentration. Springiness is a key textural attribute in drinking jellies, referring to the ability of the gel to return to its original shape after being compressed [53]. The results showed that the springiness of the drinking jelly increased with higher gelling agent concentrations, ranging from 0.81 mm to 1.24 mm. S1 had the lowest springiness, whereas S5 had the highest. A similar observation was also reported by Akesowan [50] in drinking jelly using a seaweed-based gelling agent. Furthermore, the cohesiveness of drinking jelly refers to the ability to hold together under mechanical stress and return to its original state after deformation. It is a key indicator of the internal bonding and structural integrity of the drinking jelly [54]. The cohesiveness of drinking jelly ranged from 0.42 to 0.65. The increment in GF gelling agent concentration positively affected the cohesiveness, enhancing it significantly. According to a study by Afriani [52], increasing the gelling agent concentration in jelly drinks resulted in higher cohesiveness. Additionally, gumminess and chewiness followed a similar trend, with gumminess increasing from 0.24 N to 0.42 N and chewiness from 0.31 N to 0.59 N. These changes imply that higher gelling agent concentrations result in a firmer, more cohesive, and chewier product, which is not the optimal consistency for drinking jelly. Studies have shown that a higher proportion of seaweed can lead to undesirable textures, such as increased firmness or sliminess [55,56].

### 2.6. Phytochemical and Antioxidant Properties

**Figure 7:**
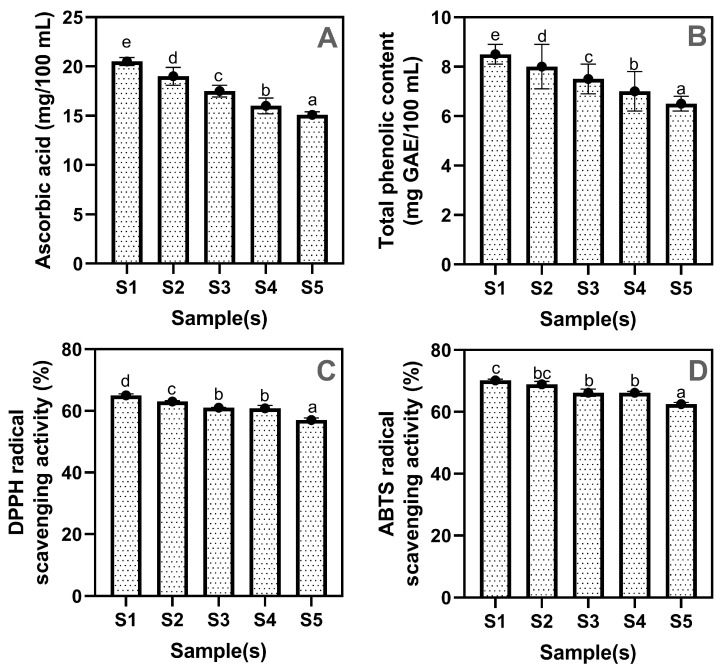
Phytochemicals (Ascorbic acid (**A**), total phenolic content (**B**)), DPPH (**C**) and ABTS (**D**) radical scavenging properties of drinking jelly made of strawberry fruit concentrate and different concentrations of GF gelling agent. Note: Different letters of the alphabet in the figure show significant differences. S1–S5 represent the drinking jelly samples prepared with varying concentrations of GF gelling agent: S1 (0.2%), S2 (0.4%), S3 (0.6%), S4 (0.8%), and S5 (1.0%).

Figure 7A–D shows the phytochemical contents and antioxidant properties of drinking jelly made with strawberry concentrate and GF gelling agent at varying concentrations. The ascorbic acid (AsA) content in the drinking jelly samples tended to decrease with the increased concentration of the GF gelling agent. This is in accordance with the study of Gubsky et al. [56]. The AsA range in the drinking jelly was observed between 20.50 mg and 15.10 mg. A higher level of AsA was found in the S1 samples, which gradually decreased as the GF concentration peaked, with S5 exhibiting the lowest AsA values among the samples. Overall, the results show a consistent decline in AsA concentration as the GF gelling agent content increases. AsA is known for its high sensitivity to oxidative conditions and environmental stressors; the structural and compositional changes induced by higher gelling agent concentrations may promote AsA degradation [57,58]. AsA content decreases in jellies with higher concentrations of seaweed gelling agents due to metal ion-catalyzed oxidation [59], thermal degradation during gelation [60], pH changes [61], and restricted diffusion in the gel network [62].

Similarly, the TPC levels in drinking jelly continuously decreased with the increased GF gelling agent concentrations. However, compared with the loss of AsA in drinking jelly, the decreased level of TPC between drinking jelly samples was minimal. Phenolic compounds generally exhibit greater stability compared to AsA, yet they remain susceptible to shifts in the food matrix that can affect their bioavailability and protective capacity [63]. TPC levels in the drinking jelly ranged between 8.50 mg and 6.50 mg. Among the samples, the S1 sample had the highest TPC level, while the S5 had the lowest TPC. Interactions between phenolics and gelling agent molecules, changes in a_w_, or subtle alterations in ionic strength may all contribute to phenolic compound losses [64]. While the decrement in TPC is less pronounced than that of AsA, it still indicates that higher gelling agent levels may not be optimal for retaining these valuable phytochemicals. On the other hand, the antioxidant properties, such as scavenging activity against DPPH and ABTS radicals, were consistent with the level of phytochemicals. As the gelling agent concentration increases in the drinking jelly compositions, the gradual loss of these key phytochemicals translates into diminished DPPH and ABTS radical-scavenging activity. Lima et al. [65] found that higher hydrocolloid concentrations in orange jellies reduced antioxidant activity, as indicated by DPPH and ABTS assays. This decline is associated with a loss of bioactive phytochemicals, such as phenolic compounds and ascorbic acid, influenced by the matrix composition and gelling agent levels. Furthermore, among the potency of radical scavenging by drinking jelly samples, the drinking jelly vastly controlled the ABTS as compared with the DPPH, and this can be concluded by their scavenging percentages, where ABTS scavenging ranged between 70.15% and 62.51%, and DPPH scavenging was between 65% and 57%. Overall, this study exhibited that the minimal concentration of GF gelling agent effectively retained more phytochemicals and antioxidant properties than the higher concentration of GF gelling agent.

### 2.7. Volatile Profile

**Figure 8:**
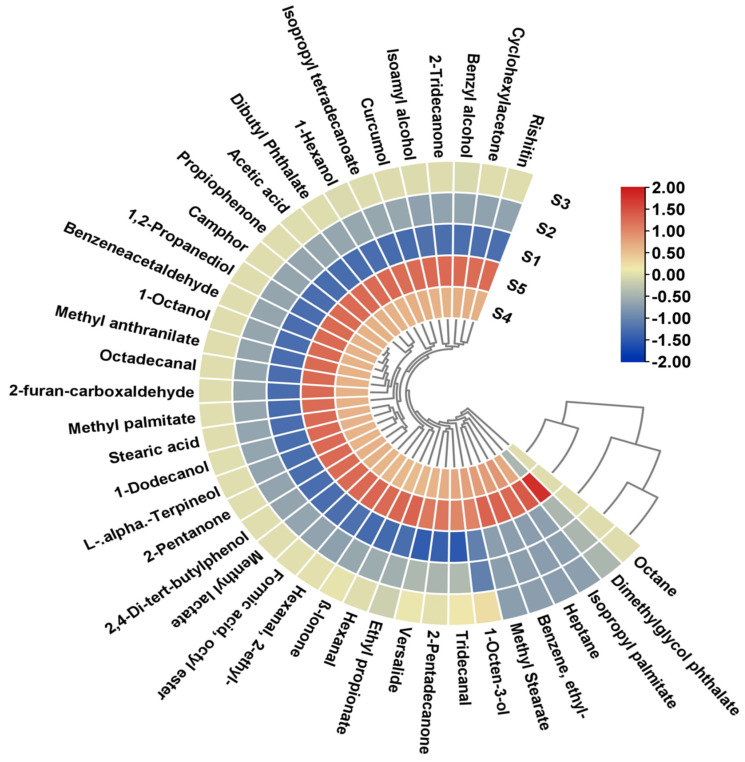
Heat map generated by TBtools showing the visualization of flavor compounds in the drinking jelly made of strawberry fruit concentrate and different concentrations of GF gelling agent. Note: S1–S5 represent the drinking jelly samples prepared with varying concentrations of GF gelling agent: S1 (0.2%), S2 (0.4%), S3 (0.6%), S4 (0.8%), and S5 (1.0%).

Table 1 provides the results of volatile compounds across strawberry-fruit-based drinking jelly samples made with varying concentrations of GF gelling agent. The heatmap (Figure 8) provides a clear visual representation of the progression and variation in the relative concentrations of 40 volatile compounds across the drinking jelly samples, complementing the detailed table data. The use of a color gradient in the heatmap, ranging from blue (low concentration) to red (high concentration), allows for quick identification of dominant and less abundant compounds, as well as trends across treatments. GC-MS analysis revealed significant enhancement in the flavor profile, primarily driven by alcohols, esters, and aldehydes, the contributions of which increased consistently with higher GF gelling agent concentrations. As shown in Table 1, the alcohol group was dominant and exhibited the most substantial increase when GF gelling agent concentration increased. 1-Hexanol and 2-ethyl displayed a near-exponential increase, rising from 9.37% in S1 to 46.86% in S5. Similarly, 1-octanol, which contributes waxy and slightly citrus-like aromas, showed significant enhancement. Isoamyl alcohol, which imparts banana-like fruity notes, gradually increased from 0.06% in S1 to 0.29% in S5. Additionally, 1-hexanol contributed to the fresh, green notes of the profile, while 1-octen-3-ol provided earthy undertones, maintaining intermediate levels throughout the progression. Esters emerged as the second-most influential group, substantially increasing from 3.84% in S1 to 16.69% in S5. Within this group, methyl anthranilate, recognized for its sweet and grape-like aroma, exhibited a notable and consistent increase, starting at 2.23% in S1 and reaching a peak of 11.16% in S5. Long-chain esters showed proportional concentration increments, including hexadecanoic acid methyl ester and heptadecanoic acid methyl ester. Ethyl propionate, contributing a distinct pineapple-like aroma and sweet-fruity notes, complemented the dominant alcohol-driven profile. The interaction between esters and the strawberry concentrate base created a synergistic fruity and sweet flavor that intensified as GF concentration increased. The aldehyde group demonstrated a significant upward trend from 1.89% in S1 to 9.55% in S5. Hexanal, known for its fresh, green aroma, rose from 0.03% to 0.17%, while octadecanal, adding waxy undertones, increased from 1.04% to 5.19%. 2,4-Dimethylbenzaldehyde significantly raised from 0.36% to 1.79%, contributing unique aromatic notes. These variations are clearly visible in the heatmap’s color progression from blue to warmer tones. Benzaldehyde and its derivatives played important roles in the overall development of the aromatic profile. Ketones exhibited mild solvent-like and sweet attributes, increasing from 0.75% in S1 to 3.85% in the S5 samples. 2-Furan-carboxaldehyde, with its caramel-like sweet aroma, rose from 0.71% to 3.57%, while 2-pentadecanone showed similar growth patterns. The increase in 1,2-propanediol from 0.27% to 1.34% suggested complex solvent interactions. Carboxylic acids, which impart sour and sharp undertones, increased from 0.59% to 2.95%, ensuring a balanced acidity in the flavor matrix. Terpenoids, recognized for their floral and citrus-like aromas, increased from 0.57% in S1 to 2.82% in S5, with L-α-terpineol significantly enhancing the floral undertones. The spicy compound rishitin maintained intermediate levels throughout the samples. Phenolic compounds increased steadily from 0.22% to 1.11%, adding subtle woody and medicinal notes that enriched the overall profile. Hydrocarbons and other compounds remained minimal throughout the progression. Chemical and solvent-like compounds, such as octane, heptane, dimethylglycol phthalate, and ethylbenzene, remained consistently low across all samples, represented by the dominance of blue hues in the heatmap. Notably, ethylbenzene emerged exclusively in the later samples (S4 and S5), suggesting threshold-dependent detectability or progressive chemical synthesis. The heatmap reveals distinct clusters of compounds, with fruity compounds showing sharp increases in S5, while earthy and spicy compounds maintained intermediate levels. This pattern highlights how dominant fruity and sweet notes overshadow the minor groups as the samples progress. The progressive enhancement in flavor complexity with increasing GF gelling agent concentrations suggests a critical role of the gelling agent in flavor retention and controlled release. GF likely improved the stability and entrapment of volatile compounds, resulting in their elevated concentrations and perceptibility in the final product. This gel-like matrix, combined with the strawberry concentrate, provided a stable base that synergized with fruity, sweet, and waxy volatiles, allowing for the optimal development of dominant sensory attributes.

## 3. Conclusions

This study comprehensively evaluated the effects of increasing GF gelling agent concentrations on strawberry-based drinking jelly’s physicochemical, antioxidant, and flavor attributes. Overall, the results demonstrate that GF gelling agent concentration profoundly influences critical quality parameters, including color characteristics, gel network structure, viscosity, a_w_, flavor profile, and phytochemical retention. Color attributes were significantly enhanced with increasing GF levels, with L* and a* values rising, while b* decreased. Optimal visual appeal was observed in the S4 samples, where the jelly achieved a balanced light red hue, enhancing its marketability. Microscopic analysis revealed that increasing GF concentrations in the drinking jelly (S1–S4) produced a homogeneous, compact gel matrix suitable for drinking jelly. However, concentrations above S4 caused excessive gelation, compromising the product’s flowability and drinkability. The physicochemical properties showed that increasing GF levels raised the pH, TSS, and TSS/TA ratio, resulting in a sweeter and less acidic product, while a_w_ decreased, improving product stability. Viscosity increased proportionally with GF concentration, enhancing the texture and mouthfeel but compromising fluidity at 1.0%. Phytochemical analysis revealed a decline in AsA and TPC as GF concentration increased, which led to reduced DPPH and ABTS radical scavenging activity, particularly at higher levels. Volatile compound analysis identified a progressive enhancement in the flavor profile, with alcohols (notably 1-octanol) and esters (such as methyl anthranilate) exhibiting the most significant increases, contributing to waxy, fruity, and sweet notes. Aldehydes such as hexanal and octadecanal complemented the dominant volatiles with fresh and grassy undertones, while terpenoids and phenolic compounds added floral and woody complexity. The GF gelling agent facilitated the entrapment and controlled release of these volatiles, resulting in the structured progression of flavor compounds. The results revealed that a GF concentration of 0.8% (S4) was optimal for producing high-quality drinking jelly. At this concentration, the product achieved desirable visual appeal, a cohesive gel structure, balanced flavor complexity, and acceptable antioxidant retention. Higher GF levels enhanced mechanical properties but led to over-gelation, compromised drinkability, and reduced phytochemical retention. This study demonstrates the efficacy of GF seaweed as a novel gelling agent for producing fruit juice-based drinking jellies, highlighting its potential in phytochemical retention. The findings expand the applications of GF seaweed beyond its traditional uses, establishing its value in modern food systems. Incorporating GF seaweed as a gelling agent improves product functionality and represents a sustainable approach to utilizing marine resources in contemporary food applications. Future research could explore how variations in GF quality or source affect its functional properties. Additionally, investigating the interaction of GF with other bioactive compounds, as well as its potential applications in diverse food matrices, could further expand its utility. Long-term storage studies would also be valuable to assess the product’s shelf life, stability, and physicochemical changes under varying storage conditions.

## 4. Materials and Methods

### 4.1. Raw Material, Chemicals, and Reagents

Fresh GF seaweed was collected from the Advanced Institute for Food Security, Prince of Songkla University, Chaiya district, Surat Thani province, Thailand. The samples were brought to the laboratory on the same day and processed immediately to ensure their quality. After cleaning to remove sand, debris, and epiphytes, the seaweed was washed thoroughly with tap water, followed by a distilled water wash. The collected seaweed was dried in a hot-air oven at 50 °C until constant weight was attained. After drying, the seaweed was milled into a fine powder using a laboratory mill (model no. PG-ECO-0300, Spring Green Evolution Co., Ltd., Bangkok, Thailand) and then stored in an airtight container at room temperature until ready for use as a gelling agent in the drinking jelly compositions. All the other ingredients used, such as strawberry concentrate, water, and citric acid, were food-grade materials. The chemicals and reagents used were methanol, ethanol, 2,2′-azino-bis(3-ethylbenzothiazoline-6-sulfonic acid (ABTS) reagent, ascorbic acid, ashless Whatman filter paper, barium chloride (BaCl_2_), pH buffer solutions (pH 4 and pH 7), 2,2-diphenyl-1-picrylhydrazyl (DPPH), Folin-Ciocalteu reagent, gallic acid, hydrochloric acid (HCl), lead acetate, methylene blue (1%), sodium carbonate (Na_2_CO_3_), sodium chloride (NaCl), and sodium hydroxide (NaOH) were purchased from LOBA Chemie Pvt. Ltd. (Mumbai, India) and Merck Ltd. (Darmstadt, Germany).

### 4.2. Drinking Jelly Preparation

**Figure 9:**
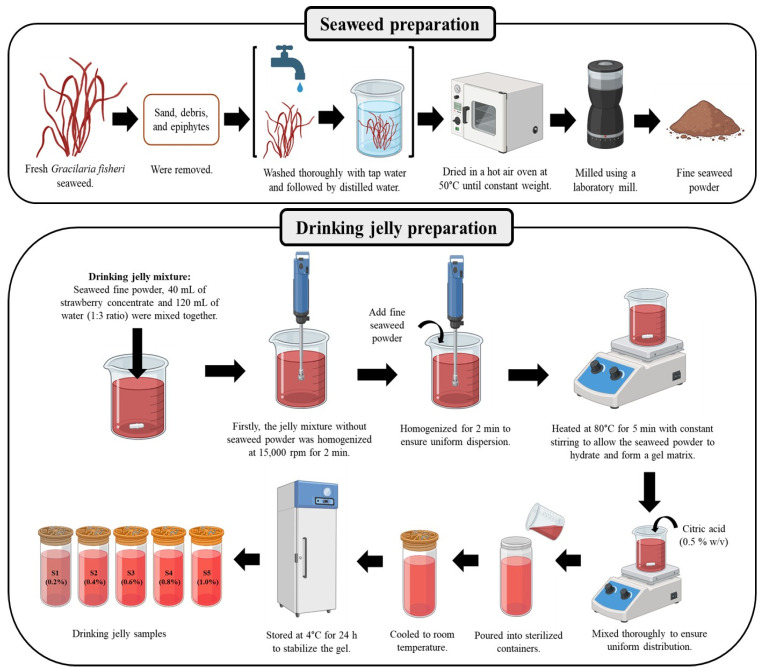
Infographic representation of GF seaweed gelling agent preparation and drinking jelly preparation using strawberry juice concentrate and different concentrations of GF gelling agent.

Drinking jelly was prepared using crude seaweed powder from GF as the gelling agent at concentrations of 0.2%, 0.4%, 0.6%, 0.8%, and 1% (*w*/*v*). Strawberry concentrate was prepared by diluting it with distilled water at a 1:3 ratio. Other ingredients included citric acid (0.5% *w*/*v*), which was used as part of the drinking jelly composition. The required amount of GF powder was weighed, and 40 mL of strawberry concentrate and 120 mL of water were combined in a beaker. The mixture was homogenized using a handheld homogenizer at 15,000 rpm for 2 min. Crude GF powder was then gradually added to the mixture with continuous stirring to prevent clumping and homogenized for another 2 min to ensure uniform dispersion. The mixture was heated on a hot plate to approximately 80 °C and maintained at this temperature for 5 min with constant stirring to allow the GF powder to hydrate and form a gel matrix. Citric acid (0.8 g) was mixed thoroughly to ensure uniform distribution. The prepared drinking jelly was poured into sterilized containers, cooled to room temperature, and stored at 4 °C for 24 h to stabilize the gel. Drinking jellies were labeled as S1 (0.2%), S2 (0.4%), S3 (0.6%), S4 (0.8%), and S5 (1.0%) based on the concentration of GF powder used. All the drinking jelly samples were measured for various physicochemical parameters, as shown in Section 2.3. Figure 9 illustrates the preparation of drinking jelly preparations.

### 4.3. Quality Analysis

#### 4.3.1. Determination of Color Characteristics and Appearance

The color characteristics of the drinking jelly, including lightness (L*), redness (a*), and yellowness (b*), were measured using a colorimeter (Hunter Lab, Reston, VA, USA). The appearance of the drinking jelly samples was also documented with a handheld digital camera (Coolpix B500, Nikon, Tokyo, Japan).

#### 4.3.2. Determination of Total Soluble Solids (TSS)

The TSS levels in the drinking jelly samples were measured by using a handheld hand refractometer (ATAGO Model PAL-1, Tokyo, Japan, Brix range 0.0–53%). The results are expressed as brix (°).

#### 4.3.3. Determination of pH and Titratable Acidity (TA)

The pH values of the drinking jelly samples were measured using a tabletop pH meter (Mettler-Toledo GmbH, Giessen, Germany), which was calibrated with pH 4 and pH 7 before use. The TA level in the drinking jelly was measured in accordance with the method of Banin et al. [66]. The results are expressed in percentage (%) citric acid.

#### 4.3.4. Determination of Viscosity

The viscosity of the drinking jelly was determined using a Brookfield Viscometer (Brookfield DVE viscometer, Middleborough, MA, USA). Samples were carefully loaded into the viscometer cup to eliminate air bubbles, and an LV-3 spindle was employed for measurement. Viscosity was recorded at a shear rate of 30 rpm under controlled temperature conditions of 25 ± 1 °C, maintained using a water bath. The results were reported in centipoise (cP).

#### 4.3.5. Determination of a_w_ and Moisture Content

The a_w_ of the drinking jelly samples was tested using the a_w_ meter (AquaLab Model series 3TE, Pullman, WA, USA). The moisture content in the drinking jelly samples was determined using a hot air oven (Binder, model FD 115, Tuttlingen, Germany) drying method (135 °C for 2 h) in accordance with the procedure of AOAC [67] (method 930.15). The moisture level in the samples was calculated using the following formula:

$$Moisture\;content\;\left(\%\right)=\frac{Loss\;of\;weight\;on\;drying}{Sample\;weight}\times 100$$

#### 4.3.6. Determination of Total Sugar and Reducing Sugar

The total sugar and reducing content in the drinking jelly were measured using the Lane and Eynon titration method as described in AOAC [67] (method 923.09). For total sugar determination, 5 mL of the sample was mixed with 3 mL of concentrated hydrochloric acid and hydrolyzed at 68 °C for 30 min to convert non-reducing sugars, such as sucrose, into reducing sugars. After cooling, the hydrolyzed sample was neutralized with 1 M sodium hydroxide, and the final volume was adjusted to 50 mL with distilled water. From this solution, 10 mL was taken and titrated against 10 mL of Fehling’s solutions A and B, which were mixed in equal volumes (5 mL each) under boiling conditions. Methylene blue (1%) was used as the indicator. The endpoint was identified as the disappearance of the blue color. The total sugar content was calculated using the standard Fehling’s factor and expressed as g per 100 g of sample. The non-reducing sugar content was determined by subtracting the reducing sugar content from the total sugar content. For reducing sugar determination, Fehling’s solutions A and B (5 mL each) were mixed, and the sample was clarified using lead acetate if necessary. A 5 mL aliquot of the prepared sample was diluted to 50 mL with distilled water. From this diluted solution, 10 mL was taken and titrated against the prepared Fehling’s solution under boiling conditions using methylene blue as the indicator. The endpoint was identified as the disappearance of the blue color. The reducing sugar content was calculated using the standard Fehling’s factor and expressed as g per 100 g of sample.

#### 4.3.7. Determination of Sulfate Content

The sulfate content of the seaweed-based gelling agent used in drinking jelly was determined using a modified method of Moses et al. [68]. An amount of 100 g of drinking jelly was thoroughly dried in an oven to a constant weight to remove all moisture. From the dried sample, approximately 1 g was weighed for analysis and hydrolyzed by boiling with 50 mL of 1 N hydrochloric acid (HCl) for 30 min to release sulfate ions from the sample matrix. After hydrolysis, 10 mL of 0.25 M barium chloride (BaCl_2_) solution was added to the hot solution to precipitate the sulfate as barium sulfate (BaSO_4_). The mixture was allowed to cool to room temperature over 5 h to ensure complete precipitation. The BaSO_4_ precipitate was collected on ashless filter paper, thoroughly rinsed with distilled water to remove impurities, and dried. The filter paper containing the precipitate was incinerated in a muffle furnace at 700 °C for 1 h to produce pure barium sulfate as white ash. After cooling, the ash residue was weighed (W_2_, in grams). The sulfate content was calculated using the following equation:

$$Sulfate\;content\;\left(mg/g\right)=\frac{W2}{W1}\times 100\times 0.4116$$

#### 4.3.8. Determination of Textural Profile

The textural profile, such as firmness, springiness, cohesiveness, gumminess, and chewiness in the drinking jelly, was measured in accordance with the method of Handayani et al. [69] with some modifications. A Texture Analyzer (LFRA 4500, Brookfield Engineering, Middleborough, UK) assessed the textural profile with a 50-mm-diameter cylindrical probe. The samples were compressed to a target distance of 5 mm at a test speed of 0.5 mm/s, with a force trigger of 5 g to ensure uniform initiation of the test. All measurements were performed at a room temperature of 25 ± 1 °C to maintain consistency.

#### 4.3.9. Determination of Ascorbic Acid

The determination of ascorbic acid content in the drinking jelly was conducted following the method of Banin et al. [66]. Drinking jelly samples were oven-dried, ground, and sieved to obtain a fine powder. A 0.025 g portion of the dried sample was mixed with 25 mL of 95% ethanol and vortexed thoroughly to ensure complete extraction. The absorbance of the extract was measured at 270 nm using a UV-Vis spectrophotometer (F-15001, Shimadzu, Kyoto, Japan). The ascorbic acid content was determined by comparing the absorbance values to a standard curve prepared with ascorbic acid solutions at concentrations of 1 μg/mL, 10 μg/mL, 20 μg/mL, 30 μg/mL, and 40 μg/mL, following the same procedure as the sample. The results were expressed in mg of ascorbic acid per 100 mL of sample.

#### 4.3.10. Determination of Total Phenolic Contents

The determination of total phenolics in drinking jelly was performed according to the method of Singleton et al. [70]. This involved adding 0.25 mL of the extracts to 2 mL of distilled water and 0.25 mL of Folin-Ciocalteu reagent, followed by 0.5 mL of 7% sodium carbonate solution. Then, the reaction mixture was thoroughly vortexed and kept in the dark for 30 min at room temperature, and then the absorbance was recorded at 730 nm using a UV-Vis spectrophotometer (F-15001, Shimadzu, Kyoto, Japan). TPC was calculated using a standard curve based on gallic acid, and the results were expressed as mg of gallic acid per 100 mL of the sample.

#### 4.3.11. Determination of Antioxidant Activities

For antioxidant activities, 10 g of drinking jelly samples were extracted with 50 mL of 80% methanol (*v*/*v*) by sonication for 30 min at room temperature. After that, the extracted mixture was centrifuged at 4000 rpm for 15 min; the supernatant was carefully collected and used for DPPH and ABTS activities. The DPPH radical scavenging activity of drinking jelly was carried out according to the method described by Brand-Williams et al. [71]. An aliquot of 100 µL of the sample was well mixed with 3.9 mL of 60 µmol/L DPPH solution in a test tube. The resulting mixture was incubated in darkness at an ambient temperature for 30 min. Then, absorbance was read at a wavelength of 515 nm by a spectrophotometer (Mini UV 1240, Shimadzu, Kyoto, Japan) and expressed as percentages. The ABTS radical scavenging activity was evaluated according to the method of Al-Momani et al. [72]. An aliquot of 0.1 mL was added to 3.9 mL of ABTS reagent and incubated for 6 min at room temperature. The absorbance was measured at a wavelength of 734 nm using a spectrophotometer (Mini UV 1240, Shimadzu, Kyoto, Japan), and the results were expressed as %.

#### 4.3.12. Determination of Volatile Profile

The volatile compounds in the drinking jelly were determined based on the method described for HS-SPME-GC/MS by Chen et al. [73], with some modifications. For the analysis, 1 g of homogenized drinking jelly was weighed into a 20 mL headspace vial, to which 0.5 mL of saturated NaCl solution was added to increase ionic strength and drive the volatiles into the headspace. The vial was sealed using a plastic cap with a polytetrafluoroethylene-silicone septum. The headspace vial was equilibrated at 60 °C and stirred at 700 rpm for 5 min to reach homogeneous volatilization. A 50/30 µm DVB/CAR/PDMS (divinylbenzene/carboxen/polydimethylsiloxane) fiber needle assembled on a 57330-U SPME handle (Supelco, Bellefonte, PA, USA) was exposed to the headspace for 50 min at 60 °C for the adsorption of volatile compounds. The fiber needle was then immediately introduced into the injection port of a 7890B gas chromatograph coupled with a 7000C mass spectrometer (Agilent Technologies, Santa Clara, CA, USA). In the injection port, volatiles were thermally desorbed at 250 °C for 3 min in split mode (3:1). The separation was performed on an HP-5MS quartz capillary column (30 m × 0.25 mm × 0.25 μm) using helium (99.999%) as the carrier gas. The flow rate of the carrier gas was 3.6 mL per min. The oven temperature was programmed as follows: initially set at 40 °C for 3 min, ramped to 180 °C at 4 °C per min, held for 2 min, and then increased to 250 °C at 10 °C per min. The electron impact ionization energy was 70 eV, the ion source and the quadrupole temperature were kept at 230 and 150 °C, respectively, and the auxiliary heating temperature was 250 °C. For the mass scanning, a range of 30 to 500 *m*/*z* was followed. Identification of volatile compounds was carried out by matching their mass spectra with the NIST 14 database and confirming their RIs. The relative concentrations of the volatile compounds in the drinking jelly samples were calculated using the peak area of each compound relative to the total peak area of all detected volatiles. The results were expressed as a % of relative concentrations.

#### 4.3.13. Microstructural Observation

The microstructural observation of drinking jelly, particularly the gel matrices and voids, was observed using a light microscope. Approximately 0.5 g of the drinking jelly was gently placed on a clean glass slide. A few drops of distilled water were added to the slide to ensure the adequate hydration and dispersion of the sample. A coverslip was then carefully placed over the specimen to create a thin and even layer and minimize the inclusion of any air bubbles. The prepared slides were subsequently placed on the microscope, and the drinking jelly was observed at a magnification of 100×. The structural characteristics of the drinking jelly matrix were documented using a handheld digital camera (Coolpix B500, Nikon, Tokyo, Japan).

### 4.4. Statistical Analysis

All experiments were carried out in triplicates, and the results were expressed as mean ± standard deviation. The data were analyzed using a completely randomized design. One-way analysis of variance was conducted using SPSS software (v12 for Microsoft Windows) to determine significant differences among treatments (*p* < 0.05). When significant differences were detected, Tukey’s Honestly Significant Difference test was used as a post hoc analysis for pairwise comparisons to identify which treatment means differed significantly.

## Figures and Tables

**Table 1:** The volatile profile of drinking jelly made of strawberry fruit concentrate and different concentrations of GF gelling agent.

RT (min)	Compound Name	CAS#	Formula	Flavor Description	Relative Concentration (%)				
					S1	S2	S3	S4	S5
1.973	Octane	589-43-5	C_8_H_18_	No distinct flavor; typically described as gasoline-like or chemical in scent.	0.08	0.08	0.08	0.08	0.08
2.858	2-Pentanone	590-86-3	C_5_H_10_O	Sweet and fruity aroma with a solvent-like note.	0.19	0.37	0.56	0.74	0.93
3.088	Ethyl propionate	2000012-94-5	C_5_H_10_O_2_	Fruity, pineapple-like flavor.	0.02	0.04	0.05	0.07	0.09
6.214	Hexanal	66-25-1	C_6_H_12_O	Green, grassy, or leafy aroma.	0.03	0.07	0.10	0.13	0.17
7.275	Benzene, ethyl-	100-41-4	C_8_H_10_	Sweet, floral, and slightly fruity aroma.	-	-	-	0.03	0.04
8.573	Heptane	590-35-2	C_7_H_16_	No distinct flavor; chemical or gasoline-like scent.	-	-	-	0.04	0.05
9.655	Hexanal, 2-ethyl-	123-05-7	C_8_H_16_O	Green and citrusy aroma.	0.04	0.07	0.11	0.14	0.18
10.779	Isoamyl alcohol	123-51-3	C_5_H_12_O	Fruity and banana-like aroma.	0.06	0.11	0.17	0.23	0.29
14.848	Cyclohexylacetone	2408-37-9	C_9_H_16_O	Mildly sweet, floral scent.	0.08	0.15	0.23	0.31	0.38
17.254	1-Hexanol	111-27-3	C_6_H_14_O	Green, woody, or leafy aroma.	0.08	0.16	0.23	0.31	0.39
22.199	Acetic acid	64-19-7	C_2_H_4_O_2_	Sharp, vinegar-like flavor.	0.25	0.51	0.76	1.02	1.27
22.249	1-Octen-3-ol	3391-86-4	C_8_H_16_O	Mushroom-like or earthy aroma.	-	-	0.05	0.07	0.08
22.424	2-furan-carboxaldehyde	98-01-1	C_5_H_4_O_2_	Sweet, caramel-like scent.	0.71	1.43	2.14	2.86	3.57
23.358	1-Octanol	104-76-7	C_8_H_18_O	Waxy, slightly citrusy aroma.	9.37	18.75	28.12	37.49	46.86
24.992	Formic acid, octyl ester	112-32-3	C_9_H_18_O_2_	Sweet, fruity aroma.	0.08	0.15	0.23	0.30	0.38
25.372	1,2-Propanediol	57-55-6	C_3_H_8_O_2_	Slightly sweet, bland taste.	2.42	4.84	7.26	9.68	12.10
25.898	Camphor	432-25-7	C_10_H_16_O	Cool, medicinal, or minty aroma.	0.13	0.26	0.39	0.52	0.65
26.308	Benzene-acetaldehyde	122-78-1	C_8_H_8_O	Floral, honey-like aroma.	0.07	0.14	0.21	0.28	0.35
27.2	L-.alpha.-Terpineol	10482-56-1	C_10_H_18_O	Lilac, floral, and slightly citrusy aroma.	0.17	0.33	0.50	0.66	0.83
27.666	Tridecanal	1604-34-8	C_13_H_26_O	Waxy, slightly citrus-like aroma.	-	0.04	0.06	0.08	0.09
28.832	Propiophenone	15764-16-6	C_9_H_10_O	Mild floral aroma.	0.36	0.72	1.08	1.44	1.79
28.997	2-Tridecanone	593-08-8	C_13_H_26_O	Slightly sweet, floral scent.	0.12	0.23	0.35	0.47	0.59
29.34	Benzyl alcohol	100-51-6	C_7_H_8_O	Sweet, floral aroma.	0.05	0.10	0.15	0.21	0.26
29.408	Menthyl lactate	77-68-9	C_12_H_24_O_3_	Cooling, minty aroma with a slight sweetness.	0.33	0.66	1.00	1.33	1.66
30.261	ß-Ionone	14901-07-6	C_13_H_20_O	Violet, floral, and woody aroma.	0.04	0.08	0.12	0.15	0.19
30.51	1-Dodecanol	112-53-8	C_12_H_26_O	Waxy, citrus-like aroma.	0.27	0.53	0.80	1.06	1.33
31.287	2-Pentadecanone	2345-28-0	C_15_H_30_O	Mild, floral, and waxy aroma.	-	0.06	0.09	0.13	0.16
31.546	Isopropyl tetradecanoate	110-27-0	C_17_H_34_O_2_	No distinct flavor; slightly oily or waxy scent.	0.08	0.17	0.25	0.34	0.42
31.724	Curcumol	10396-80-2	C_15_H_24_O_2_	Warm, woody, and slightly spicy aroma.	0.11	0.23	0.34	0.46	0.57
32.283	Octadecanal	502-69-2	C_18_H_36_O	Waxy, fatty aroma.	1.04	2.08	3.11	4.15	5.19
32.996	Methyl anthranilate	134-20-3	C_8_H_9_NO_2_	Grape-like, sweet aroma.	2.23	4.46	6.69	8.93	11.16
33.185	Methyl palmitate	112-39-0	C_17_H_34_O_2_	Waxy, fatty scent.	0.17	0.35	0.52	0.69	0.86
33.498	Isopropyl palmitate	142-91-6	C_19_H_38_O_2_	Oily, waxy scent.				0.02	0.03
33.7	2,4-Di-tert-butylphenol	96-76-4	C_14_H_22_O	Mild, medicinal, or phenolic scent.	0.22	0.44	0.67	0.89	1.11
34.014	Versalide	88-29-9	C_18_H_26_O	Musky and slightly floral aroma.		0.04	0.06	0.08	0.10
34.348	Stearic acid	1731-92-6	C_18_H_36_O_2_	Waxy, fatty scent.	0.34	0.67	1.01	1.34	1.68
36.024	Methyl Stearate	5129-61-3	C_19_H_38_O_2_	Waxy, fatty aroma.	-	-	-	0.03	0.04
39.724	Dibutyl Phthalate	84-74-2	C_16_H_22_O_4_	Chemical, slightly sweet scent.	0.23	0.47	0.70	0.94	1.17
39.738	Dimethylglycol phthalate	117-82-8	C_14_H_18_O_6_	Chemical, slightly sweet aroma.	0.70	0.70	0.70	0.70	0.88
41.044	Rishitin	1020-31-1	C_14_H_22_O_2_	Woody, earthy, or slightly spicy aroma.	0.12	0.23	0.35	0.47	0.58

Note: S1–S5 represent the drinking jelly samples prepared with varying concentrations of GF gelling agent: S1 (0.2%), S2 (0.4%), S3 (0.6%), S4 (0.8%), and S5 (1.0%).

## Data Availability

The data presented in this study are openly available in article.
